# Complications Following Orchiectomy in Stallions in Field Conditions: Descriptive Results and Predictors in a Study of 612 Cases

**DOI:** 10.3390/ani16020326

**Published:** 2026-01-21

**Authors:** Panagiota Tyrnenopoulou, Eugenia Flouraki, Leonidas Folias, Epameinondas Loukopoulos, Alexandros Starras, Panagiotis Chalvatzis, Vassiliki Tsioli, Vasia S. Mavrogianni, George C. Fthenakis

**Affiliations:** 1 Veterinary Faculty, University of Thessaly, 43100 Karditsa, Greece; ptyrnenop@uth.gr (P.T.); eflouraki@uth.gr (E.F.); eloukopoulos@uth.gr (E.L.); vtsioli@vet.uth.gr (V.T.); vmavrog@vet.uth.gr (V.S.M.); 2 Private Veterinary Practice, 41110 Larissa, Greece; leonfolias@gmail.com; 3 Private Veterinary Practice, 73100 Chania, Greece; starrasvet@gmail.com; 4 Private Veterinary Practice, 50100 Kozani, Greece; chalvatzispanagiotisvet@hotmail.com

**Keywords:** castration, horse, orchiectomy, post-surgery complication, stallion, testis

## Abstract

The objectives of this work were (i) to evaluate the incidence of complications in male horses after orchiectomy performed in the field, i.e., away from a veterinary hospital, (ii) to describe the post-operative complications that occurred in these animals and (iii) to study the potential predictors for the development of such complications. This study involved 612 animals, on which orchiectomy was performed. The operative part of the study was carried out by one of three experienced veterinary surgeons. Standard pre-operative procedures were followed. Orchiectomy was performed using one of three principal surgical techniques, open, semi-closed or closed, and hemostasis was achieved by means of one of the various procedures, specifically the use of the Henderson instrument, the use of the Reimer emasculator, the ligation of the testicular artery or combinations thereof. In cases of reported complications, these were verified and identified appropriately. At least one (any) post-operative complication was recorded in 145 horses (23.7%). The most frequently observed complication was scrotal swelling/seroma formation, which was observed in 130 animals (21.2% of all animals; 89.7% of animals with at least one (any) complication). Another six different complications were observed, specifically colic, continued stallion-like behavior, evisceration, funiculitis, hemorrhage and scrotal infection. The median age of horses with complications was significantly older than that of animals with no complications: 11 versus 9 years. Animals in which the open technique was applied developed post-operative complications more frequently (30.1% of animals thus operated); further, complications were observed more frequently in animals on which operations were performed by using the Henderson instrument (84.6% of animals) versus animals on which any of the various other procedures were applied (22.4%). In multivariable analysis, the following predictors emerged for the development of complications: the surgical technique employed, the procedure applied for hemostasis, older animal age, and heavier animal bodyweight. The findings confirm that orchiectomy in stallions, even when performed in field conditions, is, in general, a safe procedure. The identification of predictors suggests that veterinarians should take additional care when planning to operate on animals at higher risk or when using surgical approaches that increase the potential for the development of complications.

## Simple Summary

The present work describes complications that occurred after orchiectomy performed in the field in male horses, as well as potential predictors for the development of such complications. The study is based on a dataset of 612 cases in male horses, operated in conditions of general veterinary practice by one of three experienced veterinary surgeons. At least one post-operative complication was recorded in 24% of the horses. The wide array of complications diagnosed in these animals included scrotal swelling/seroma formation, most often, and, less frequently, colic, continued stallion-like behavior, evisceration, funiculitis, hemorrhage and scrotal infection. This study identified that older or heavier horses, as well as horses operated by means of the open surgical technique or with the use of the Henderson instrument for achieving hemostasias, were at a higher risk of developing complications. The findings confirm that orchiectomy in horses, even when performed in field conditions, is, in general, a safe procedure. Veterinarians should take additional care when planning to operate on animals at higher risk or when using surgical approaches that increase the potential for the development of complications.

## 1. Introduction

In stallions, orchiectomy (‘castration’) is a surgical procedure frequently performed by veterinarians active in equine veterinary practice. It is undertaken with the aims to manage undesirable behavior in animals, to reduce their aggression and to prevent reproductive activity [1,2]. Despite the evaluation of non-surgical alternatives, for example anti-GnRH immunization [3,4], which may offer reversible suppression of reproductive function, orchiectomy remains a mainstay approach in equine practice, due to its reliability, predictability and consistent outcomes. Although it is considered as a routine operation, several post-operative complications have been reported; these may range from mild to severe scrotal edema, potentially life-threatening events, e.g., hemorrhage, evisceration or septic funiculitis [5,6,7,8]. Such complications adversely affect the welfare of the operated animals and also have financial consequences for the owners [9]. The development and severity of these complications may be influenced by various factors, which can be animal-related factors or factors associated with the surgical procedure [2].

Often, and depending on the circumstances (e.g., difficulty to transport the stallion to a veterinary hospital), the procedure is carried out in field conditions, i.e., on the premises where an animal lives. Such cases present unique challenges compared to cases involving animals handled in hospital-based settings. These include the maintenance of aseptic conditions and the prevention of potential environmental contamination, as well as the correct post-operative supervision of the animal [10]. A clear understanding of factors that can be associated with complications after orchiectomy performed in field conditions is important, in order to improve surgical outcomes.

Previous relevant studies have focused on work carried out in controlled clinical settings and have presented information regarding descriptive findings and factors potentially associated with complications therein. Nevertheless, relevant information regarding the situation in orchiectomy surgeries performed in field conditions remains limited.

The present retrospective study–analysis presents the post-operative complications identified from a comprehensive dataset of orchiectomies performed on stallions in field conditions. The specific objectives of this work were (i) to evaluate the incidence of complications in male horses after orchiectomy performed in field conditions, i.e., away from a veterinary hospital, (ii) to describe the post-operative complications that occurred in these animals and (iii) to study the potential predictors for the development of such complications. This study–analysis includes 612 animals, on which orchiectomy had been performed in the field by three veterinary surgeons in Greece.

## 2. Materials and Methods

### 2.1. Cases Included in This Study–Analysis

The results of orchiectomy performed on 612 male horses were considered in this retrospective study–analysis. The median age of the horses, which was recorded based on the identification document of each animal, was 9 years (interquartile range (IQR): 9 years). The median bodyweight of the animals, which was recorded by using a measuring tape, was 380 kg (IQR: 230 kg). In total, horses were of 11 different breeds, most frequently mixed breeds (30.7% of all animals) and Shetland ponies (18.3%) (Table S1). All the animals were privately owned and were living at purpose-built horse barns, often with field access.

The criteria for considering stallions for orchiectomy under field conditions (and thus for inclusion in the analysis) were (a) a horse age of at least two years, (b) good clinical conditions (as established through a general, detailed, standardized physical examination of the animal pre-operatively), (c) the presence of both testes within the scrotum (as established by means of palpation) and (d) the absence of any previous attempt of orchiectomy. The criteria for excluding the animals for orchiectomy under field conditions were (a) clinical signs of ill health, (b) unilateral or bilateral cryptorchidism, as well as any other clinically evident testicular abnormalities, and (c) previous attempt(s) of orchiectomy.

### 2.2. Surgeons

The operative part of this study was carried out by one of three experienced veterinary surgeons. All three surgeons had been active in equine veterinary practice in different parts of Greece, with over five years of relevant clinical experience. Each of the three surgeons contributed a different number of cases to the study (*n* = 364, *n* = 81, *n* = 187), in accordance with the respective clinical load.

### 2.3. Pre-Operative Procedures

Standard pre-operative procedures were followed by all three surgeons. These included (a) confirmation of anti-tetanus vaccination in the horses or, if animals had not been previously vaccinated, the instigation of a full vaccination regime six weeks prior to the planned operation, (b) restraint from mating activity one month before the operation, (c) detailed general clinical examination, as well as examination of the genital system one day prior to the planned operation, and (d) fasting twelve hours before the operation (two hours before the operation for water fasting).

Immediately before the start of the operation, all animals were administered a broad-spectrum antibiotic combination (penicillin and streptomycin (Penicilline Streptomycine 200/200^®^, MSD Animal Health, Rahway, NJ, USA) with a dose rate of 8 mg procaine benzylpenicillin and 8 mg dihydrostreptomycin per kg bodyweight [11,12]).

### 2.4. Analgesic–Anesthetic Procedures

For orchiectomy in the standing position, horses were sedated via the intravenous administration of detomidine (20–40 µg per kg bodyweight (bw)) and butorphanol (10 µg per kg bw). Following the standard pre-surgical preparation, local analgesia was performed with aseptic infiltration of 10 mL 2% lidocaine into each testis.

Orchiectomy in recumbency was performed under general anesthesia. Premedication consisted of intravenous administration of (a) romifidine (60–80 µg per kg bw) with butorphanol (10 µg per kg bw) or (b) detomidine (20–40 µg per kg bw) with butorphanol (10 µg per kg bw). Anesthesia was induced via intravenous administration of midazolam (0.1 mg per kg bw) and ketamine (2.5 mg per kg), with horses subsequently positioned in left lateral recumbency. Following standard pre-surgical preparation, local analgesia was provided by aseptic infiltration into the testis, as described hereabove.

### 2.5. Surgical Procedures

Orchiectomy was performed using one of three principal surgical techniques: open, semi-closed or closed. The surgical approach to each testis was performed through a separate scrotal incision. In general, the surgical part of the work was performed as detailed by Schumacher [1], Baldwin [10], Moll et al. [13], Carmalt et al. [14] and Kilcoyne et al. [15]; the techniques followed are outlined below.

- Open Orchiectomy Technique: In the open technique, the parietal tunic was incised and left open following removal of the testis. The incision of the tunic exposed the caudal ligament of the epididymis, on which a transection was performed, in order to release the testis and epididymis. These structures were removed using an emasculator. This technique required minimal dissection and could be performed in standing sedated animals or in recumbent animals under anesthesia. However, as the parietal tunic had been incised, there was potential for communication with the abdominal cavity [1,8,15].
- Semi-Closed Orchiectomy Technique: The semi-closed technique involved initial dissection of the parietal tunic from the scrotal fascia, followed by an incision through the tunic to allow exteriorization of the testis, epididymis, and a portion of the spermatic vasculature and cord. The spermatic vasculature was then either ligated or emasculated before transection. The parietal tunic could be independently crushed, transected, or suture-closed [2,15].
- Closed Orchiectomy Technique: In the closed technique, the parietal tunic was dissected free from the scrotal fascia, but was not incised. The intact tunic was removed together with the testis and epididymis. Hemostasis and closure of the tunic were achieved by the crushing action of an emasculator or by applying a ligature [1,10,15].

At the end of the surgical procedure, hemostasis was achieved by means of one of various procedures, specifically the use of the Henderson instrument, the use of the Reimer emasculator, the ligation of the testicular artery or combinations thereof. The methods employed for hemostasis are briefly described below.

- Use of The Henderson Instrument: The use of the Henderson instrument involved drill-activated equipment that grasped and twisted the spermatic cord, in order to achieve hemostasis through torsion rather than crushing or ligation. The rapid rotation resulted in controlled tearing and sealing of the vessels, reducing the likelihood of post-operative hemorrhage. Notably, the use of the instrument required careful alignment of the cord and consistent rotational speed, in order to produce effective vascular occlusion [1,5,15,16].
- Ligation of The Testicular Artery: During ligation of the testicular artery, the spermatic cord was isolated and the testicular artery was secured with an absorbable ligature prior to transection of the cord. The ligation provided direct mechanical control of the arterial flow and was often selected in horses in which the potential for an increased risk of post-operative hemorrhage had been determined, e.g., older animals. After ligation of the artery, the spermatic cord was transected approximately 2 cm distally to the ligature using Mayo scissors or a scalpel blade (no. 10 or no. 22), depending on surgeon preference, while avoiding excessive residual tissue [1,10].
- Ligation of The Testicular Artery with Concurrent Inguinal Ring Suturing: Rarely, ligation of the testicular artery was combined with suturing of the inguinal ring. This additional step was performed in order to reduce the risk of evisceration in horses with a large external inguinal ring or a suspected predisposition to herniation. After vascular ligation, the superficial inguinal ring was partially closed using absorbable sutures to minimize potential communication with the abdominal cavity [1,5].
- Use of The Reimer Emasculator. The Reimer emasculator provided a two-step mechanism, where crushing and cutting of the spermatic cord occurred separately. The jaws of the emasculator first crushed the cord to occlude the vasculature, and after complete crushing had occurred, the cutting blade was activated to sever the cord. This way, effective hemostasis was achieved by allowing a longer compression phase before the transection. In this method, correct instrument orientation and the allowance of adequate time to fully crush the spermatic cord were essential to prevent post-operative bleeding [1,17].
- Use of The Reimer Emasculator with Ligation of The Testicular Artery: Occasionally, the Reimer emasculator was used in conjunction with ligation of the testicular artery. This procedure enhanced hemostatic control in horses deemed to be at higher risk of excessive post-operative hemorrhage. After ligation of the artery, the emasculator was applied distally to the ligature, with the aim to sever the spermatic cord, while reinforcing vascular occlusion through the application of dual hemostatic mechanisms [1,17].

At the end, the scrotal incision was left open to heal by second intention.

The choice for the surgical technique and the approach for hemostasis applied in each operation was decided by the operating surgeon and was influenced by individual animal characteristics, perceived intra-operative risk, equipment availability and surgeon preference.

### 2.6. Post-Operative Care

Subsequent to the completion of the surgery, all animals received flunixin meglumine (1.1 mg per kg bw), administered via intravenous injection, followed by repeat daily administrations (at least three days) at the same dose [18].

Animals were monitored by the owners every 2 h for the first 12 h post-operatively, and subsequently, every 6 h for the following five days. All animals were restricted within a box for 24 h post-operatively to minimize movements; they were allowed to walk at a mild pace in paddocks on the second day after operation, guided and restrained by their owners. Moreover, cold-hosing of the genital area was carried out twice daily for five days post-operatively.

Pharmaceutical treatment included the continuation of pre-surgical antibiotic administration for five days after the surgery.

Animals were routinely re-examined by the surgeons who operated on them on the day after the operation, as well as 9 to 12 days later. Finally, general advice was given to avoid interaction of the operated stallions with mares for at least two months after surgery.

### 2.7. Development of Surgical Complications

In cases of observed complications, which were reported by the owners, a visit was carried out by the surgeon who operated on the horse within 12 h. In such cases, a detailed clinical examination was performed. The surgical complications were verified, identified, and then recorded appropriately.

The final outcome for the animals in which complications were observed was recorded.

### 2.8. Data Management and Analysis

Data were entered into Microsoft Excel. Basic descriptive analyses were performed and exact binomial confidence intervals were obtained.

The outcome ‘development of a complication after orchiectomy’ was considered. In total, seven independent variables (Table S2) were evaluated for association with this outcome in univariable analyses, by using Pearson’s chi-square test or the Mann–Whitney test, as appropriate.

Subsequently, a multivariable model was constructed. Independent variables found with *p* < 0.20 in the preceding univariable analyses were offered to the model. Variables were progressively removed from the model by using backward elimination, with *p* > 0.20 employed as the threshold value; among those found with *p* > 0.20, the one with the largest *p* was removed from the model. The procedure was repeated until no value could be removed from the model, i.e., with *p* > 0.20. The variables included in the final multivariable model constructed are in Table S2. The existence of multicollinearity between the variables that were used in the multivariable analysis (*n* = 5) for the development of a complication after orchiectomy was assessed by calculating the variance inflation factors between each of these variables. Moreover, the associations between these five variables were subsequently evaluated using principal component analysis. The multivariable analysis was repeated thrice by taking into account within each model only the animals operated on by each of the three surgeons. The variables included in each of the final multivariable models constructed are in Table S3.

Thereafter, the outcomes ‘development of colic after orchiectomy’, ‘development of funiculitis after orchiectomy’ and ‘development of scrotal infection after orchiectomy’, which are three serious post-operative complications, were considered. The procedures described hereabove (assessment in univariable and multivariable analyses) were performed for each of these three outcomes for specific complications after orchiectomy. The variables included in each of the final multivariable models constructed are in Table S3.

Statistical significance was defined at *p* < 0.05.

## 3. Results

### 3.1. Frequency and Description of Post-Operative Complications

#### 3.1.1. Frequency and Type of Post-Operative Complications

At least one (any) post-operative complication was recorded in 145 horses (23.7%, 95% confidence interval (CI): 20.5–27.2%)). The most frequently observed complication was scrotal swelling/seroma formation, which was observed in 130 animals (21.2% of all animals, 95% CI: 18.2–24.7%; 89.7% of animals with at least one (any) complication). Another six different complications were observed in the horses in this study; these were colic, continued stallion-like behavior, evisceration, funiculitis, hemorrhage and scrotal infection (Table 1).

Notably, in 36 animals (24.8% of those with complications), two different complications were observed, and in 3 animals (2.1% of those with complications), three different complications were observed.

#### 3.1.2. Associations with Characteristics of the Animals

**Figure 1:**
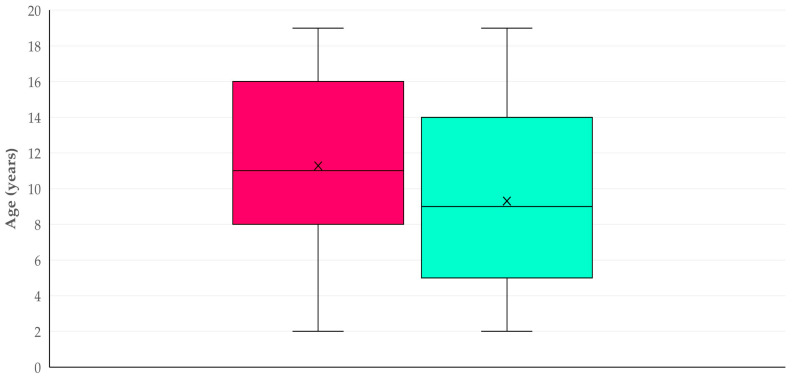
Box and whisker plot of the age of stallions in which post-orchiectomy complications were (red) or were not (green) observed.

The median age of horses with complications was significantly older than that of animals with no complications: 11 (IQR: (8) years versus 9 (IQR: 9) years (*p* < 0.0001) (Figure 1). However, no difference in median age was seen between horses, in which only one or at least two complications were seen: 11 (IQR: 9) years for both cohorts (*p* = 0.40).

The median bodyweight of horses with complications did not differ significantly from that of animals with no complications: 380 (IQR: 220) kg versus 400 (IQR: 220) kg (*p* = 0.10). Also, no significant association was found between the bodyweight class of the animals and the development of post-orchiectomy complications: 22.0% of horses had a bodyweight ≤ 300 kg, 20.3% of horses had a bodyweight between 300 and 450 kg and 28.6% of horses had a bodyweight of over 450 kg (*p* = 0.11). Further, no difference in median bodyweight was seen between horses in which only one or at least two complications were seen: it was 400 (IQR: 190) kg for animals with one complication versus 390 (IQR: 250) kg for animals with two or three complications (*p* = 0.07).

Although the development of complications was more frequently seen in Lippizaner (100%, i.e., one animal) and Pura Raza Espanola (66.7% out of six animals), the differences between horse breeds were not significant (*p* = 0.10).

#### 3.1.3. Associations with the Surgeon Who Performed the Orchiectomy

No significant differences in the proportions of horses observed with complications were evident among animals operated on by each of the three surgeons who participated in the study: these proportions were 24.4%, 17.3% and 25.1%, respectively (*p* = 0.34). Also, there were no significant differences between the three surgeons in the proportions of horses with at least two complications, which were 4.9%, 11.1% and 7.2%, respectively (*p* = 0.10).

#### 3.1.4. Associations with Surgical Methodologies Employed

There was no significant difference in the proportions of animals that did or did not develop complications as a consequence of the operation performed with the animal in the standing or recumbent position: these proportions were 22.0% and 23.9%, respectively (*p* = 0.75).

**Figure 2:**
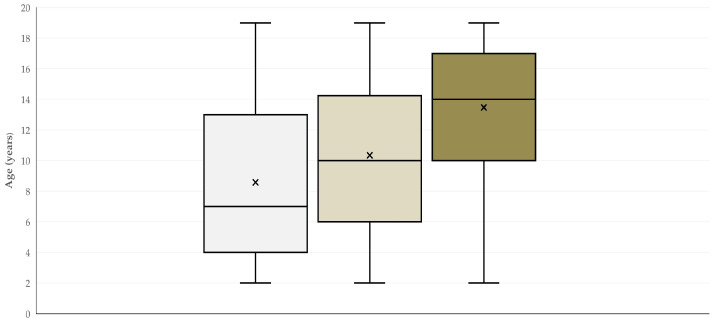
Box and whisker plot of the age of stallions, in accordance with the surgical technique employed for orchiectomy—(from left to right) light grey plot: horses in which the open technique was applied; dark gray plot: horses in which the semi-closed technique was applied; brown plot: horses in which the closed technique was applied.

There was a significant difference in the proportion of horses that developed complications as a result of the technique employed. Specifically, animals in which the open technique was applied developed complications more frequently (Table 2) (*p* < 0.0001), although horses on which that technique was used were significantly younger (median age: 7 (IQR: 9) years). Although horses operated on with the semi-closed or the closed technique were of older age (9 (IQR: 7.5) years and 14 (IQR: 7) years, respectively; Figure 2) (*p* < 0.0001 between the three cohorts), they developed complications significantly less often (*p* < 0.0001) (Table 2).

Moreover, complications were observed more frequently in operations in which hemostasis was attempted by applying the Henderson instrument, specifically 84.6% of animals versus 22.4% among animals in which any of the various other procedures were applied (Table 3) (*p* < 0.0001). No significant difference was evident in the median age between the two cohorts of horses (10 (IQR:7) versus 9 (IQR: 9) years, respectively; *p* = 0.24).

### 3.2. Predictors

#### 3.2.1. Predictors for at Least One (Any) Post-Operative Complication

The results of the univariable analyses are in Table S4.

**Figure 3:**
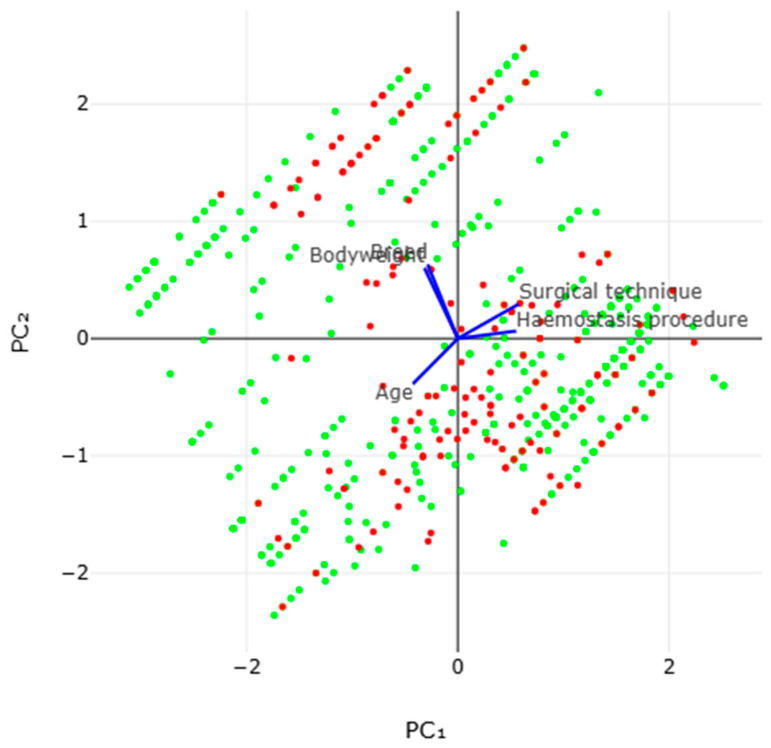
Biplot of results of principal component analysis for the development (red) or lack of development (green) of a complication post-orchiectomy in horses (vectors clockwise from the top right: surgical technique employed, procedure applied for hemostasis, age of animal, bodyweight class of animal, breed of animal) (standard scaling, with no rotation during preprocessing; blue colour lines indicate the loading vectors).

In the multivariable analysis, four predictors were identified for the development of a complication after orchiectomy. These were (a) the surgical technique employed (*p* < 0.0001), (b) the procedure applied for hemostasis (*p* < 0.0001), (c) animals of older age (*p* < 0.0001) and (d) animals of a heavier bodyweight class (≥450 kg) (*p* = 0.014) (Table 4). Variance inflation factors between the five variables in the final multivariable model varied from 1.00008 to 1.27755 (Table S5). The principal component analysis of the development of a complication indicated that the two principal components accounted for 61.3% of the variation (Figure 3 and Figure S1 and Table 5).

In the multivariable analyses performed by taking into account only the animals contributed by each of the three surgeons, two predictors consistently emerged to be significant for the development of complications. These were (a) the surgical technique employed (*p* ≤ 0.002) and (b) the procedure applied for hemostasis (*p* ≤ 0.015). Additionally, older age emerged as a significant predictor in cases in which two of the surgeons performed the operation (*p* ≤ 0.004) (Table S6).

#### 3.2.2. Predictors for Specific Serious Complications

The results of the univariable analyses are in Table S7.

In the multivariable analysis, the following predictors were identified for the development of specific serious complications after orchiectomy. For the development of colic, these were (a) the surgical technique employed (*p* = 0.003) and (b) the procedure applied for hemostasis (*p* = 0.012), and for the development of funiculitis, these were (a) the surgical technique employed (*p* = 0.002) and (b) older age (*p* = 0.012) (Table S8). For the development of scrotal infection, no significant predictor emerged (*p* > 0.015 for all evaluations).

### 3.3. Final Outcome of Complications

Only one animal with a post-operative complication (specifically, evisceration) was euthanized (0.7% (95% CI: 0.1–3.8%), based on welfare grounds, as this was deemed to be beyond treatment; thus, mortality was 0.2% (95% CI: 0.0–0.9%) overall. All the other animals (*n* = 144) recovered fully.

## 4. Discussion

### 4.1. Preamble

The present study provides extensive information regarding potential adverse outcomes of stallion orchiectomies, taking into consideration a large and diverse sample of animals. Notably, the present study refers to performing the operations in field conditions rather than in veterinary hospitals, which can lead to increased risk for the development of complications.

Previous studies were restricted to evaluating complications in surgeries performed in hospital-based settings, where control of the surgical procedures and monitoring of the patient were optimal [15]. In contrast, the present work reflects conditions in general equine veterinary practice and highlights the practical and clinical importance of evaluating orchiectomy outcomes under true field conditions. The large number of cases included in this study and the involvement of three different surgeons (between whom no significant differences were identified) provided a foundation for establishing benchmark results regarding a surgical procedure performed frequently in equine veterinary practice. Indeed, orchiectomy is frequently performed by veterinarians offering an ambulatory service, often with limited assistance and variable resources. Therefore, it is essential to show that the frequency of post-operative, serious, life-threatening complications is low, even under these circumstances, with the aim to reinforce the confidence of the demanding clientele.

No randomization procedure was followed by the operating surgeons during selection of surgical technique and procedure for hemostasis, which might have resulted in some bias among independent variables for each target outcome. This became evident in the strong association between the age of animals and the surgical technique employed by the surgeons, which clearly reflected their effort to prevent the development of post-operative complications in older animals by choosing a technique associated with fewer incidents in the international literature. However, the nature of the present study, which is a retrospective analysis, did not allow for such a design, as the three veterinarian–surgeons, who contributed cases in the present analysis, primarily intended to perform the operation with no post-operative complications and applied the relevant methodologies that they considered most appropriate, based on their personal clinical experience and the relevant literature, explaining the strong association between the age of animals and the surgical technique employed detected in this dataset. In any case, no multicollinearity emerged among the independent variables, as the calculated variance inflation factors were low (<1.3), which indicated that it was negligible to very low.

### 4.2. Development of Post-Operative Complications

Although the overall rate of observed post-operative complications was around 25%, in most of the horses in which adverse events occurred, these were self-limiting. Scrotal swelling/seroma formation were observed more frequently than any other complication (in approximately 90% of animals with adverse events), whilst serious complications were noted in less than 2% of animals operated on. These figures corroborate the reports that severe morbidity following castration may occur in only a few animals [2,5,8].

The complete lack of tetanus development reflects the consistent and correct application of relevant vaccination protocols in the animals, whilst the low incidence of infections post-operatively indicates that hygiene measures during surgery were adequate, particularly when coupled with the administration of effective antibacterial drugs. Although the routine use of peri-operative antibiotics for clean elective surgery remains debatable [19], there are reports indicating that systemic administration of antimicrobials in orchiectomy cases in field conditions could contribute to reduced inflammatory markers and infections, and, thus, could be practiced to mitigate relevant risks inherent to such environments [11,20].

These findings should be considered in conjunction with the conditions in which the surgeries were carried out. Given the field conditions in which the orchiectomies were performed, frequent post-operative monitoring of the animals by their owners was applied as a standard practice, and, moreover, the surgeon also examined the animals on the day following the operation, with a repeat visit within one-and-a-half weeks. We postulate that the frequent post-operative monitoring, firstly, contributed to identifying all animals in which complications occurred, and, secondly, contributed to the rapid response in cases of animals with post-operational complications, which resulted in only one loss and low mortality in the remaining animals, lower than that reported internationally [9,15]. Thus, the findings highlight the importance of post-operative monitoring and care in field environments, where hygiene conditions can be difficult to maintain.

### 4.3. Identification of Predictors

Predictors identified for the potential development of post-operative complications included animal-related and surgery-related variables. The identification of predictors can guide pre-operative planning and would allow veterinarians to anticipate and mitigate risks through case selection, technique modification and close monitoring.

Among the former variables, older age was associated with a higher risk of complications than younger age; this may have occurred as a result of the increased number of matings among older horses compared to younger horses.

The median age of the horses on which orchiectomy was performed in the present study–analysis (9 years) was greater than that of horses reported to have undergone this operation in other countries; examples include the United Kingdom (age range of animals: 2 to 8 years) [9] and the United States of America (median age of animals: 12 months) [15]. This gap reflects regional differences in the characteristics of animal populations. Horses in Greece are maintained mostly for mixed use, predominantly leisure, and may be presented for orchiectomy at an older age, frequently following the development of undesirable stallion behavior. In contrast, data from the aforementioned countries mostly involved sport-horse populations, e.g., thoroughbred racehorses, which were managed under intensive training and operated on in hospital-based settings.

Moreover, bodyweight was found to be important as well; the increased incidence found in heavier animals likely reflects increased testicular size, vascular diameter and connective tissue density, which have been reported to predispose horses to intra-operative hemorrhage or the delayed healing of wounds [10].

The use of the open technique for orchiectomy was found to be associated with a significantly higher incidence of complications after orchiectomy, and it can be associated with the exposure of the vaginal tunic of the animals. This approach may potentially facilitate and contribute to infection of the surgical area [2].

Despite the precautions taken by the three surgeons, who selected the closed technique for use in older animals, thus avoiding the convergence of two potential risk factors in the same surgical case, older age and the use of the open technique both nevertheless emerged as significant predictors of the development of complications. This underlines their importance and strong weighting in the development of post-orchiectomy complications.

The use of the Henderson instrument for the application of hemostasis was associated with a significantly higher incidence of post-operative complications, which may have resulted from the synergistic adverse effect of older age and use of the procedure, as reported by Hinton et al. [21], who found that its application in animals older than four (4) years increased the risk of surgical complications. This procedure potentially offered less consistent control over individual vascular structures, which may have contributed to higher complication rates when compared with those when techniques based on crushing (e.g., use of a Reimer emasculator) or ligation were used. These two approaches provided direct mechanical occlusion of the testicular vasculature and were, therefore, considered to achieve more predictable hemostasis [2,17]. Comino et al. [17] observed more efficient hemostatic capacity after applying the Reimer emasculator when using the open surgical technique, a finding with which ours cannot be compared. Overall, the present findings have underscored the importance of using hemostatic procedures that maximize vascular control, with minimum tissue disruption, particularly in field settings in which post-operative monitoring of the animals could be limited.

## 5. Conclusions

This paper has provided an updated reference for expected adverse outcomes of orchiectomy on stallions. Overall, the consistency of these outcomes across different populations of animals and independently of the surgeon who operated on each stallion points to the reliability of performing orchiectomy in field conditions. The present findings confirm that orchiectomy in stallions, even when performed in field conditions, is, in general, a safe procedure. The identification of predictors for the development of complications after orchiectomy suggests that veterinarians should take additional care when planning to operate on animals at higher risk or when using surgical approaches that increase the potential for the development of complications.

## Figures and Tables

**Table 1:** Frequency of complications observed in 612 stallions after orchiectomy.

Post-Operative Complication Observed	Number of Stallions, in Which the Complication Was Observed	Proportion Among Stallions, in Which Complications Were Observed
Colic	11	7.6%
Continued stallion-like behavior	9	6.2%
Evisceration	1	0.7%
Funiculitis	15	10.3%
Hemorrhage	5	3.4%
Scrotal infection	16	11.0%
Scrotal swelling/seroma formation	130	89.7%

**Table 2:** Proportion of stallions in which complications were observed after orchiectomy, in accordance with the surgical technique employed.

Surgical Technique Employed	Number of Stallions, in Which the Technique Was Used	Proportion of Stallions, in Which Complications Were Observed
Open	445	30.1%
Semi-closed	26	0.0%
Closed	141	7.8%

**Table 3:** Proportion of horses in which complications were observed after orchiectomy, in accordance with the procedure applied for hemostasis.

Hemostasis Procedure Applied	Number of Stallions, in Which the Procedure Was Applied	Proportion of Stallions, in Which- Complications Were Observed
Use of the Henderson instrument	13	84.6%
Ligation of the testicular artery	278	25.5%
Ligation of the testicular artery with concurrent inguinal ring suturing	1	0.0%
Use of the Reimer emasculator	298	19.1%
Use of the Reimer emasculator with ligation of the testicular artery	22	27.3%

**Table 4:** Predictors for the development of a complication after orchiectomy in horses.

Variables	Odds Ratio	*p* Value
Surgical Technique Employed		<0.0001
Open (134/445, 30.1% ^1^)	22.88 (1.38–378.28 ^2^)	0.029
Semi-closed (0/26, 0.0%)	reference	---
Closed (11/141, 7.8%)	4.67 (0.27–81.73)	0.29
Hemostasis Procedure Applied		<0.0001
Use of the Henderson instrument (11/13, 84.6% ^1^)	23.25 (5.01–107.83 ^2^)	0.0001
Ligation of the testicular artery (71/207, 25.5%)	1.45 (0.98–2.15)	0.07
Ligation of the testicular artery with concurrent inguinal ring suturing (0/1, 0.0%)	1.40 (0.06–34.81)	0.83
Use of the Reimer emasculator (57/241, 19.1%)	reference	---
Use of the Reimer emasculator with concurrent ligation of the testicular artery (6/16, 27.3%)	1.59 (0.59–4.23)	0.36
Age of Animal		0.0001
Per unit (year) increase	1.01 (1.01–1.02 ^2^)	0.0001
Bodyweight Class of Animal		0.014
<300 kg (44/193, 22.8% ^1^)	1.21 (0.75–1.95 ^2^)	0.44
300 kg–450 kg (41/209, 19.6%)	reference	---
≥450 kg (60/210, 28.6%)	1.64 (1.04–2.58)	0.033

^1^ Proportion of animals that showed the variable of interest; ^2^ 95% confidence interval.

**Table 5:** Eigenvalues for principal component analysis for the development of complications post-orchiectomy in horses.

Parameter	PC_1_	PC_2_	PC_3_	PC_4_	PC_5_
Eigenvalue	1.80	1.27	0.78	0.68	0.48
% of variance	36.0	25.3	15.5	13.7	9.5
Cumulative (%)	36.0	61.3	76.8	90.5	100.0

## Data Availability

The data presented in this study are available on request from the corresponding author.

## Supplementary Materials

The following supporting information can be downloaded at https://www.mdpi.com/article/10.3390/ani16020326/s1: Figure S1: Scree plot of results of principal component analysis for the development of complications after orchiectomy in horses; Table S1: Breeds of 612 horses that underwent orchiectomy and were assessed for evaluation of complications; Table S2: Independent variables used for assessment of possible associations with complications following orchiectomy in stallions; Table S3: Details of multivariable models employed for the evaluation of possible associations with complications following orchiectomy in stallions; Table S4: Results of univariable analyses for associations of independent variables with development of a complication after orchiectomy; Table S5: Variance inflation factors calculated between the five variables in the final multivariable model for predictors for the development of a complication after orchiectomy in horses; Table S6: Predictors for the development of a complication after orchiectomy in horses, described separately in accordance with the surgeon who performed the operations; Table S7: Results of univariable analyses with significant associations of independent variables with development of specific complications after orchiectomy; Table S8. Predictors for the development of specific serious complications after orchiectomy in horses.
