# Supplementary material for: Complications Following Orchiectomy in Stallions in Field Conditions: Descriptive Results and Predictors in a Study of 612 Cases
Source: Animals (Basel). 2026 Jan 21;16(2):326. doi: 10.3390/ani16020326 (PMC12837701; doi:10.3390/ani16020326)
Supplement: Supplementary file 1 [file animals-16-00326-s001.zip › animals-4085686-supplementary.pdf]

Complications Following Orchiectomy in Stallions in Field Conditions: Descriptive Results and Predictors in a Study of 612 Cases

P. Tyrnenopoulou, E. Flouraki, L. Folias, E. Loukopoulos, A. Starras, P. Chalvatzis, V. Tsioli, V.S. Mavrogianni, and G.C. Fthenakis

Table S1. Breeds of 612 horses that undertook orchiectomy and were assessed for evaluation of complications.

Horse breed	No. of horses included in the analysis
Arabian	13 (2.1%)
Cob	9 (1.5%)
Haflinger	91 (14.9%)
Lippizaner	1 (0.2%)
Mix breed	188 (30.7%)
Pura Raza Española	6 (1.0%)
Shetland pony	112 (18.3%)
Skyrian	19 (3.1%)
Standardbred	1 (0.2%)
Thoroughbred	80 (13.1%)
Warmblood	92 (15.0%

Table S2. Independent variables used for assessment of possible associations with complications following orchiectomy in stallions.

Independent variables	
1.	Surgeon who performed the operation (one of three surgeons)
2.	Age of animal under operation (continuous)
3.	Bodyweight of animal under operation (≤ 300 kg, 301-450 kg, > 450 kg)
4.	Breed of animal under operation (Arabian, Cob, Halinger, Lippizaner, Mix breed, Pura Raza Española, Shetland pony, Skyrian, Standardbred, Thoroughbred, Warmblood)
5.	Position of animal during operation (standing, recumbent)
6	Surgical technique employed ('closed', 'semi-closed', 'open')
7.	Procedure applied for haemostasis (use of the Henderson instrument, ligation of the testicular artery, ligation of the testicular artery with concurrent inguinal ring suturing, use of the Reimer emasculator, use of the Reimer emasculator with ligation of the testicular artery)

Table S3. Details of multivariable models (n = 4) employed for the evaluation of possible associations with complications following orchiectomy in stallions.

Outcome	Variables required in the final multivariable model
development of a complication after orchiectomy	Age of animal under operation, Bodyweight of animal under operation, Breed of animal under operation, Surgical technique employed, Procedure applied for haemostasis
development of a complication after orchiectomy – surgeon A	Age of animal under operation, Bodyweight of animal under operation, Breed of animal under operation, Surgical technique employed, Procedure applied for haemostasis
development of a complication after orchiectomy – surgeon B	Age of animal under operation, Surgical technique employed, Procedure applied for haemostasis
development of a complication after orchiectomy – surgeon C	Age of animal under operation, Bodyweight of animal under operation, Breed of animal under operation, Surgical technique employed, Procedure applied for haemostasis
development of colic after orchiectomy	Surgical technique employed, Procedure applied for haemostasis
development of funiculitis after orchiectomy	Age of animal under operation, Breed of animal under operation, Surgical technique employed,
development of scrotal infection after orchiectomy	Bodyweight of animal under operation

Table S4. Results of univariable analyses for associations of independent variables with development of a complication after orchiectomy.

	Stallions that developed complicatios [1]	Stallions that did not develop complication(s) [1]	*p* value
Surgeon who performed the operation			
Surgeon A	89/364 = 24.5%	275/364 = 75.5%	0.34
Surgeon B	14/81 = 17.3%	67/81 = 82.7%	
Surgeon C	42/167 = 25.1%	125/167 = 74.9%	
Age of animal under operation			
Age of animal	median = 11 (IQR: 8) years	median = 9 (IQR: 9) years	< 0.0001
Bodyweight class of animal under operation			
≤ 300 kg	46/209 = 22.0%	163/209 = 78.0%	0.11
301-450 kg	39/193 = 20.2%	154/193 = 79.8%	
> 450 kg	60/210 = 28.6%	150/210 = 71.4%	
Breed of animal under operation			
Arabian	3/13 = 23.1%	10/13 = 76.9%	0.10
Cob	2/9 = 22.2%	7/9 = 77.8%	
Haflinger	27/91 = 29.7%	64/91 = 70.3%	
Lippizaner	1/1 = 100.0%	0/1 = 0.0%	
Mix breed	36/188 = 19.1%	152/188 = 80.9%	
Pura Raza Española	4/6 = 66.7%	2/6 = 33.3%	
Shetland pony	23/112 = 20.5%	89/112 = 79.5%	
Skyrian	7/19 = 36.8%	12/19 = 63.2%	
Standardbred	0/1 = 0.0%	1/1 = 100.0%	
Thoroughbred	20/80 = 25.0%	60/80 = 75.0%	
Warmblood	22/92 = 23.9%	70/92 = 76.1%	
Position of animal during operation			
Standing	13/59 = 22.0%	46/59 = 78.0%	0.75
Recumbent	132/553 = 23.9%	421/553 = 76.1%	
Surgical technique employed for the operation			
open	134/445 = 30.1%	311/445 = 69.9%	< 0.0001
semi-closed	0/26 = 0.0%	26/26 = 100.0%	
closed	11/141 = 7.8%	130/141 = 92.2%	
Procedure applied for haemostasis			
Use of the Henderson instrument	11/13 = 84.6%	2/13=15.4%	< 0.0001
Ligation of the testicular artery	71/278=25.5%	207/278=74.5%	
Ligation of the testicular artery with concurrent inguinal ring suturing	0/1=0.0%	1/1=100.0%	
Use of the Reimer emasculator	57/298 = 19.6%	241/298 = 80.4%	
Use of the Reimer emasculator with ligation of the testicular artery	6/22 = 27.3%	16/22 = 72.7%	

[1] No. of horses (proportion among horses with the trait of interest).

Table S5. Variance Inflation Factors calculated between the five variables in the final multivariable model for predictors for the development of a complication after orchiectomy in horses.

	Age of animal under operation	Bodyweight of animal under operation	Breed of animal under operation	Surgical technique employed	Procedure applied for haemostasis
Age of animal under operation					
Bodyweight of animal under operation	1.000076				
Breed of animal under operation	1.000762	1.06718			
Surgical technique employed	1.163535	1.016315	1.000514		
Procedure applied for haemostasis	1.042015	1.022581	1.023682	1.277546	

Figure S1. Scree plot of results of principal component analysis for the development of complications after orchiectomy in horses.

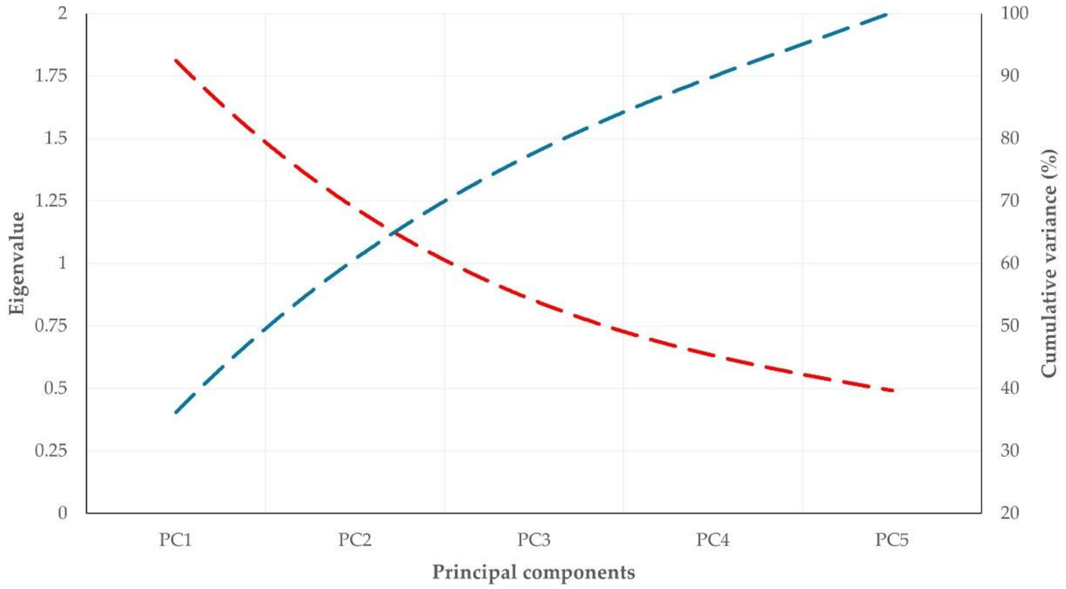

Table S6. Predictors for the development of a complication after orchiectomy in horses, described separately in accordance with the surgeon who performed the operations.

Variables	Odds ratio	*p* Value
Surgeon A		
Surgical technique employed		< 0.0001
open (81/271, 29.9% [1])	12.41 (0.73-210.48 [2])	0.08
semi-closed (0/14, 0.0%)	reference	---
closed (8/79, 10.1%)	3.45 (0.19-63.13)	0.40
Age of animals		< 0.0001
Per unit (year) increase	1.02 (1.01-1.03)	< 0.0001
Procedure applied for haemostasis		< 0.0001
Use of the Henderson instrument (4/5, 80.0% [1])	32.00 (1.56-656.09)	0.025
Ligation of the testicular artery (47/165, 28.5%)	3.19 (0.39-26.18)	0.28
Ligation of the testicular artery with concurrent inguinal canal suturing (0/1, 0.0%)	1.89 (0.05-72.03)	0.73
Use of the Reimer emasculator (37/184, 20.1%)	2.01 (0.24-16.61)	0.52
Use of the Reimer emasculator with concurrent ligation of the testicular artery (1/9, 11.1%)	reference	---
Bodyweight of animals		0.015
< 300 kg (27/129, 20.9% [1])	reference	---
300 kg – 450 kg (22/103, 21.4%)	1.03 (0.54-1.93)	0.94
≥ 450 kg (40/132, 30.3%)	1.64 (0.93-2.89)	0.08
Surgeon B		
Surgical technique employed		0.002
open (12/57, 21.1% [1])	2.53 (0.52-12.42 [2])	0.25
semi-closed (0/3, 0.0%)	1.11 (0.04-28.53)	0.95
closed (2/21, 9.5%)	reference	---
Age of animals		0.004
Per unit (year) increase	1.02 (1.01-1.03)	0.041
Procedure applied for haemostasis		0.015
Use of the Henderson instrument (3/3, 100.0% [1])	45.89 (2.02-1044.27)	0.016
Ligation of the testicular artery (4/33, 12.1%)	reference	---
Ligation of the testicular artery with concurrent inguinal canal suturing (0/0)	n/r [3]	---
Use of the Reimer emasculator (7/43, 16.3%)	1.41 (0.38-5.29)	0.61
Use of the Reimer emasculator with concurrent ligation of the testicular artery (0/2, 0.0%)	1.31 (0.05-31.98)	0.87
Surgeon C		
Surgical technique employed		< 0.0001
open (41/117, 35.0% [1])	21.58 (2.86-162.73)	0.003
semi-closed (0/9, 0.0%)	1.42 (0.05-37.68)	0.83
closed (1/41, 2.4% [1])	reference	---
Procedure applied for haemostasis		0.002
Use of the Henderson instrument (4/5, 80.0% [1])	17.85 (1.84-173.15)	0.013
Ligation of the testicular artery (20/80, 25.0%)	1.49 (0.68-3.26)	0.32
Ligation of the testicular artery with concurrent inguinal canal suturing (0/0)	n/r [3]	---
Use of the Reimer emasculator (13/71, 18.3%)	reference	---

Use of the Reimer emasculator with concurrent ligation of the testicular artery (5/11, 45.5%)	3.72 (0.98-14.07)	0.05

[1] No. of horses (proportion among horses with the trait of interest); [2] 95% confidence interval; [3] not relevant.

Table S7. Results of univariable analyses with significant associations of independent variables with development of specific complications after orchiectomy.

	Stallions that developed complicatios [1]	Stallions that did not develop complication(s) [1]	*p* value
Development of colic after orchiectomy			
Breed of animal under operation			
Arabian	0/13 = 0.0%	13/13 = 100.0%	0.001
Cob	1/9 = 11.1%	8/9 = 88.9%	
Haflinger	0/91 = 0.0%	91/91 = 100.0%	
Lippizaner	0/1 = 0.0%	1/1 = 100.0%	
Mix breed	4/188 = 2.1%	182/188 = 97.9%	
Pura Raza Española	0/6 = 0.0%	6/6 = 100.0%	
Shetland pony	1/112 = 0.9%	111/112 = 99.1%	
Skyrian	3/19 = 15.8%	16/19 = 84.2%	
Standardbred	0/1 = 0.0%	1/1 = 100.0%	
Thoroughbred	1/80 = 1.3%	79/80 = 98.7%	
Warmblood	1/92 = 1.1%	91/92 = 98.9%	
Procedure applied for haemostasis			
Use of the Henderson instrument	2/13 = 15.4%	11/13=84.6%	0.004
Ligation of the testicular artery	5/278=1.8%	273/278=98.2%	
Ligation of the testicular artery with concurrent inguinal ring suturing	0/1=0.0%	1/1=100.0%	
Use of the Reimer emasculator	3/298 = 1.0%	295/298 = 99.0%	
Use of the Reimer emasculator with ligation of the testicular artery	1/22 = 4.5%	21/22 = 95.5%	
Development of funiculitis after orchiectomy			
No variables found with significant associations			
Development of scrotal infection after orchiectomy			
Breed of animal under operation			
Arabian	0/13 = 0.0%	13/13 = 100.0%	0.049
Cob	0/9 = 0.0%	9/9 = 100.0%	
Haflinger	2/91 = 2.2%	89/91 = 97.8%	
Lippizaner	0/1 = 0.0%	1/1 = 100.0%	
Mix breed	5/188 = 2.7%	183/188 = 97.3%	
Pura Raza Española	1/6 = 0.0%	5/6 = 100.0%	
Shetland pony	1/112 = 0.9%	111/112 = 99.1%	
Skyrian	0/19 = 0.0%	19/19 = 100.0%	
Standardbred	0/1 = 0.0%	1/1 = 100.0%	
Thoroughbred	0/80 = 0.0%	80/80 = 100.0%	
Warmblood	7/92 = 7.6%	85/92 = 92.4%	

[1] No. of horses (proportion among horses with the trait of interest).

Table S8. Predictors for the development of specific serious complications after orchiectomy in horses.

Variables	Odds ratio	*p* Value
Development of colic		
Surgical technique employed		0.003
open (11/445, 2.5% [1])	7.49 (0.44-127.92 [2])	0.16
semi-closed (0/26, 0.0%)	5.34 (0.10-275.08)	0.40
closed (0/141, 0.0%)	reference	---
Procedure applied for haemostasis		0.012
Use of the Henderson instrument (2/13, 15.4% [1])	14.42 (2.18-95.34 [2])	0.006
Ligation of the testicular artery (5/207, 2.4%)	1.96 (0.46-8.32)	0.36
Ligation of the testicular artery with concurrent inguinal canal suturing (0/1, 0.0%)	22.71 (0.78-660.60)	0.07
Use of the Reimer emasculator (3/241, 1.2%)	reference	--
Use of the Reimer emasculator with concurrent ligation of the testicular artery (1/16, 6.3%)	5.29 (0.52-53.96)	0.16
Development of funiculitis		
Surgical technique employed		0.002
open (15/445, 3.4% [1])	10.19 (0.61-171.39 [2])	0.11
semi-closed (0/26, 0.0%)	5.34 (0.10-275.08)	0.40
closed (0/141, 0.0%)	reference	---
Age of animals		0.012
Per unit (year) increase	1.01 (0.99-1.02)	0.17

[1] No. of horses (proportion among horses with the trait of interest); [2] 95% confidence interval.
